# Design of multi-target activity landscapes that capture hierarchical activity cliff distributions

**DOI:** 10.1186/1758-2946-4-S1-P4

**Published:** 2012-05-01

**Authors:** Dilyana Dimova, Jürgen Bajorath

**Affiliations:** 1 Department of Life Science Informatics, B-IT, LIMES Program Unit Chemical Biology and Medicinal Chemistry, Rheinische Friedrich-Wilhelms-Universität, Dahlmannstr. 2, D-53113 Bonn, Germany

## 

For compounds with activity against multiple targets the ensuing multi-target structure-activity relationships (mtSARs) are generally difficult to analyze. However, understanding mtSARs is often of critical importance for compound design and optimization [1]. In addition, the detection and analysis of activity cliffs also plays a crucial role in comprehensive SAR exploration [2,3]. Activity cliffs represent the most prominent feature of activity landscapes, which can be graphically represented by models that integrate molecular similarity and potency relationships [2,4]. Different activity landscape representations have been introduced. These activity landscape designs have in common that they all focus on activity against a single or at most two biological targets (the latter case giving rise to selectivity landscapes [3]). For compounds active against multiple targets, landscape representations cannot be obtained directly on the basis of currently available models and new design concepts are required. Here we introduce a methodology to derive and visualize multi-target activity landscapes and systematically analyze activity cliff distributions [5]. The framework is based on a general activity cliff classification scheme. Multi-target activity landscapes are visualized as graphs where nodes represent individual compounds and edges activity cliffs. In addition, node proximity indicates molecular similarity. The methodology has been applied to derive landscape models for various compound data sets with activity against multiple targets belonging to different families. The resulting representations identify single and multi-target activity cliffs and reveal hierarchical cliff distributions. From these landscape models, compounds forming complex activity cliffs can be readily selected.
